# Data on alveolar mandibular bone thickness in Class I skeletal patient with bimaxillary protrusion

**DOI:** 10.1016/j.dib.2021.107423

**Published:** 2021-10-07

**Authors:** Rizki Andika Putra Siregar, Hilda Fitria Lubis, Muslim Yusuf

**Affiliations:** a Orthodontics Specialist Program, Faculty of Dentistry, Universitas Sumatera Utara, Medan, Indonesia; b Department of Orthodontics, Faculty of Dentistry, Universitas Sumatera Utara, Medan, Indonesia

**Keywords:** Alveolar bone thickness, Bimaxillary protrusion, Symphisis mandibular, Lateral cephalographs

## Abstract

The alveolar bone thickness influences both diagnosis and limitation of tooth movement, therefore significance retraction was commonly applied in treating patients with bimaxillary protrusion. This is a retrospective data collection of pre and post treatment lateral cephalographs from 18 to 40 years old patient treated with four premolars extraction. The alveolar mandibular bone thickness was identified in sagittal planes with Image-J software based on cephalometry lateral radiographs. Statistical analysis namely Wilcoxon test and Pearson correlation analysis coefficient were used to understand the correlation of alveolar mandibular bone thickness variables and mandibular incisors position to skeletal profile treated with first premolars extraction are presented. This data is essential for advancing in a further understanding of Class I skeletal patients with bimaxillary protrusion.

## Specifications Table

**Table:** 

Subject	Clinical research
Specific subject area	Dental
Type of data	Figure, Table
How data were acquired	Alveolar mandibular bone thickness was evaluated in sagittal planes with Image-J software based on cephalometry lateral radiographs.
Data format	Raw and analyzed
Parameters for data collection	Patients between 18 and 40 years old, that were diagnosed as Class I skeletal with bimaxillary protrusion and treated with first premolar extraction and standard edgewise mechanotherapy
Description of data collection	Data were collected using pre and post treatment lateral cephalometry radiographs that was diagnosed as Class I skeletal with bimaxillary protrusion who were treated in Orthodontics department of Dental Hospital Universitas Sumatera Utara during 2010 to 2020.
Data source location	Department of Orthodontics, Faculty of Dentistry, Universitas Sumatera Utara, Medan, Indonesia.
Data accessibility	Repository name: Mendeley Data Data identification number: https://data.mendeley.com/datasets/vm623jvvfs/1

## Value of the Data

• These data provide the differences in mandibular symphysis alveolar bone before and after mandibular anterior incisor retraction in Class I malocclusion with bimaxillary protrusion patients that is common in Asian population.
• These data are benefit to dental practitioners and researchers from understanding the bimaxillary protrusion with skeletal Class I relationships, which showed limited alveolar bone thickness and density from the cervical to the apical regions. The bone remodelling and response to the mechanism towards orthodontic forces in alveolar mandibular bone thickness related to orthodontic treatment modality.
• The data could helps to determine the retraction magnitude and alveolar bone thickness assessment used lateral cephalometric radiography regardless of unclear structure possibilities due to two dimensional film characters, thus the of main information could be obtained from lateral cephalometric imaging with low radiation in some emerging countries.

## 1 Data Description

In this data, the reliability test for alveolar mandibular bone thickness was performed by the same operator and after 10 days Cronbach's alpha showed value of 0.895, which is within the range of previous report [1,2]. It indicated a high level of internal consistency for our scale with this specific sample. The data obtained were subjected to a normality test using the Shapiro-Wilk test, however the data that was not normally distributed, the differences of alveolar mandibular bone thickness before and after retraction was calculated using the Wilcoxon test [3] as shown in Table 1. In addition, the correlation of alveolar mandibular bone thickness variables after orthodontic treatment with Spearman analysis was summarized in Table 2. The full raw data of the patients can be found in supplementary material.

**Table 1:** Differences of alveolar mandibular bone thickness before and after retraction.

		Mean ± SD (mm)			
Variable	N	Before treatment	After treatment	Differences	*p-value*
C’ – C	22	4.93 ± 1.37	4.26 ± 1.03	0.67 ± 0.65	0.000*
P – B		6.11 ± 4.13	5.41 ± 3.05	0.70 ± 2.82	0.236
B’ - P’		5.44 ± 3.33	4.90 ± 2.13	0.54 ± 2.70	0.903
S – A		8.17 ± 3.86	7.86 ± 3.53	0.30 ± 1.59	0.200
S’ – A		7.63 ± 2.18	7.25 ± 2.34	0.38 ± 1.18	0.277
I – NB		6.86 ± 3.21	3.84 ± 1.77	3.02 ± 2.21	0.000*

Note:* p<0.05: significant difference.

The result of statistical analysis showed that there was a significant difference (p<0.01) in alveolar bone thickness at the C'-C and I-NB point measurement, while the other variables were not found to be significant. The most extensive differences was found in incisors position to skeletal profile (I-NB), which is 3.02 ± 2.21 mm.

**Table 2:** Correlation of alveolar mandibular bone thickness variables after orthodontic treatment in Class I skeletal with bimaxillary protrusion.

Variables	*p-value*	*R*
C’- C	0.000*	0.702
P – B	0.000*	0.753
B’- P’	0.000*	0.706
S – A	0.000*	0.775
S’- A	0.000*	0.784

Note:* significant correlation.

The evaluation of mandibular symphysis alveolar bone thickness in Class I skeletal with bimaxillary protrusion showed significant correlation in all variables between before and after treatment based on lateral cephalometric radiographs. There were a strong, positive correlation in alveolar mandibular bone thickness between before and after treatment for all variables, which were statistically significant (r = 0.702∼0.787 and *p* = 0.000)

## 2 Experimental Design, Materials and Methods

The retrospective study used pre and post-treatment lateral cephalometry radiographs of patients that was diagnosed as Class I skeletal with bimaxillary protrusion and treated with first premolar extraction and standard edgewise mechanotherapy during 2010–2020 in Orthodontics department of Dental Hospital Universitas Sumatera Utara. There were some conditions in medical records that will be excluded, such as: if the range of patient ages was not between 18 and 40 years old while the pre and post treatment lateral cephalographs was taken, agenesis, metabolic bone disease history, cleft or lip palate, severe periodontal disease, lower incisor trauma history, and patients undergoing orthopedic or orthognatic history.

**Fig. 1:**
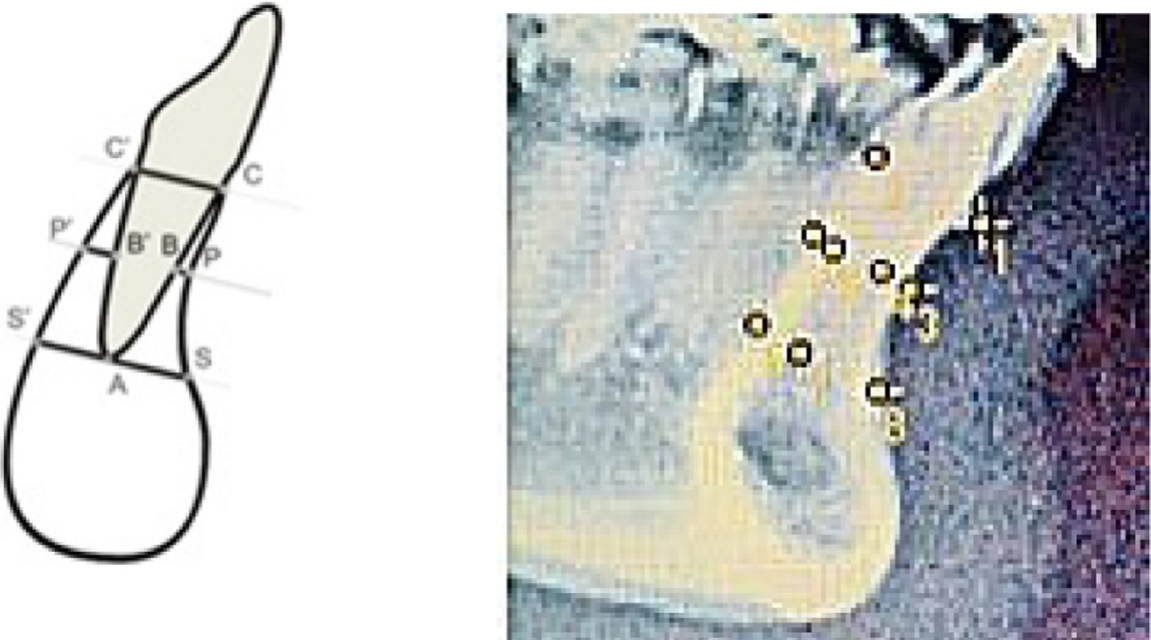
Landmark to measure mandibular symphysis thickness.

Fig. 1 depicts the alveolar mandibular bone thickness was evaluated by the following landmarks [4]:

1. C-C' = Distance from alveolar process to mandibular symphysis at labial and lingual crest level.
2. P-B = Labial bone at the middle of mandibular incisor root. From middle of the root to most labial area of mandibular incisor to external edge of labial cortical mandibular symphysis.
3. P'-B' = Lingual bone at the middle of mandibular incisor root to the most labial area of mandibular incisor to external edge of lingual cortical mandibular symphysis.
4. S-A = Labial bone at apical mandibular incisor to the most labial area of mandibular incisor to external edge of labial cortical mandibular symphysis.
5. S'-A = Lingual bone at apical mandibular incisor to the most labial area.

**Fig. 2:**
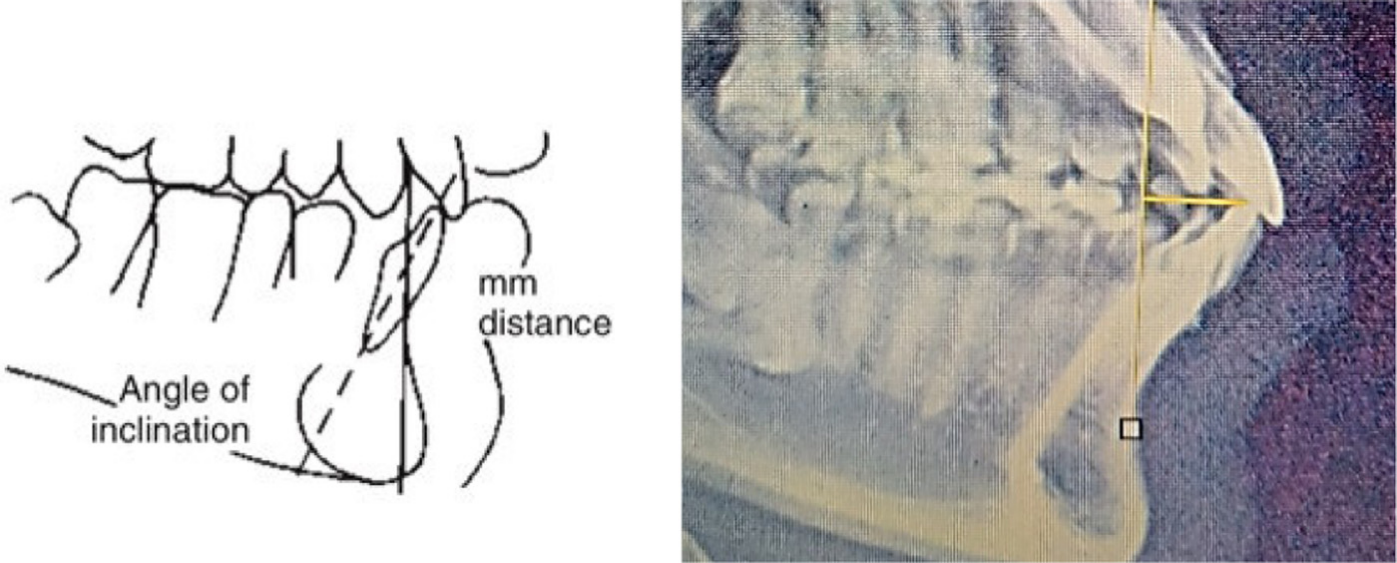
Landmarks to measure mandibular incisor retraction.

Fig. 2 showed mandibular incisors position to skeletal profile that was evaluated by mandibular incisor (I) point and NB (Nasion to B point) line. Those landmark points were determined in pre and post treatment lateral cephalometry using Image-J software.

### 2.1 Statistical analysis

The data obtained in this study were subjected to a normality test using the Shapiro-Wilk test (p<0.05) prior to analysis. Since the data that was not normally distributed, the differences of alveolar mandibular bone thickness before and after retraction (BR and AR) were calculated using the Wilcoxon test with significant difference 0.05. Then analysis the correlation of alveolar mandibular bone thickness variables after orthodontic treatment with Spearman test (p<0.05) [5].

## Ethics Statement

The authors kept to all ethical concerns during the data gathering process. The authors ensured that all respondents information used for research purposes only and confidential with the approval from Ethical Comittee from Universitas Sumatera Utara (No. 273/KEP/USU/2020).

## CRediT authorship contribution statement

**Rizki Andika Putra Siregar:** Conceptualization, Methodology, Investigation, Writing – original draft. **Hilda Fitria Lubis:** Supervision, Writing – review & editing. **Muslim Yusuf:** Validation, Writing – review & editing.

## Declaration of Competing Interest

The authors declare that they have no known competing financial interests or personal relationships which have or could be perceived to have influenced the work reported in this article.

## Appendix Supplementary materials

Image, application 1
